# 1,2-Bis(2-hy­droxy-5-methyl­benzyl­idene)hydrazine

**DOI:** 10.1107/S160053681302148X

**Published:** 2013-08-07

**Authors:** B. Saravanan, A. Jayamani, N. Sengottuvelan, G. Chakkaravarthi, V. Manivannan

**Affiliations:** a Centre for Research and Development, PRIST University, Vallam, Thanjavur 613 403, India; b Department of Chemistry, DDE, Alagappa University, Karaikudi 630 003, India; c Department of Physics, CPCL Polytechnic College, Chennai 600 068, India

## Abstract

The mol­ecular structure of the title compound, C_16_H_16_N_2_O_2_, is stabilized by intra­molecular O—H⋯N hydrogen bonds with *S*(6) graph-set motifs, so that the mol­ecule is almost planar, with a C=N—N=C torsion angle of −179.7 (2)° and a dihedral angle of 1.82 (12)° between the aromatic rings. In the crystal, weak C—H⋯π inter­actions lead to the formation of a three-dimensional network.

## Related literature

For the biological activity of Schiff base ligands, see: Kelley *et al.* (1995 ▶); Pandeya *et al.* (1999 ▶); Singh & Dash (1988 ▶); Tarafder *et al.* (2002 ▶). For standard bond lengths, see: Allen *et al.* (1987 ▶). For related strucutures, see: Chantrapromma *et al.* (2010 ▶); Fun *et al.* (2010 ▶). For graph-set motifs, see: Bernstein *et al.* (1995 ▶).
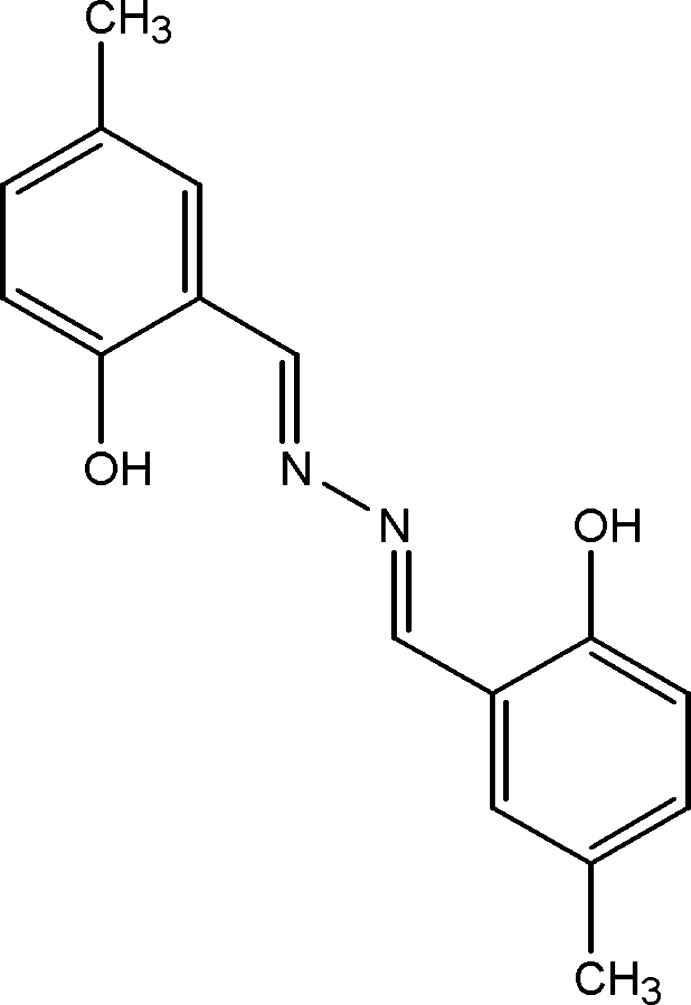


## Experimental

### 

#### Crystal data

C_16_H_16_N_2_O_2_
*M* *_r_* = 268.31
Orthorhombic, 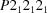

*a* = 6.0108 (5) Å
*b* = 7.3394 (5) Å
*c* = 31.674 (2) Å
*V* = 1397.32 (17) Å^3^
*Z* = 4
Mo *K*α radiation
μ = 0.09 mm^−1^
*T* = 295 K
0.22 × 0.18 × 0.16 mm

#### Data collection

Bruker Kappa APEXII diffractometer
Absorption correction: multi-scan (*SADABS*; Sheldrick, 1996 ▶) *T*_min_ = 0.982, *T*_max_ = 0.987
5699 measured reflections
2952 independent reflections
1780 reflections with *I* > 2σ(*I*)
*R*_int_ = 0.023

#### Refinement

*R*[*F* ^2^ > 2σ(*F* ^2^)] = 0.050
*wR*(*F* ^2^) = 0.154
*S* = 1.02
2952 reflections
185 parameters
H-atom parameters constrained
Δρ_max_ = 0.17 e Å^−3^
Δρ_min_ = −0.17 e Å^−3^

### 

Data collection: *APEX2* (Bruker, 2004 ▶); cell refinement: *SAINT* (Bruker, 2004 ▶); data reduction: *SAINT*; program(s) used to solve structure: *SHELXS97* (Sheldrick, 2008 ▶); program(s) used to refine structure: *SHELXL97* (Sheldrick, 2008 ▶); molecular graphics: *PLATON* (Spek, 2009 ▶); software used to prepare material for publication: *SHELXL97*.

## Supplementary Material

Crystal structure: contains datablock(s) global, I. DOI: 10.1107/S160053681302148X/is5296sup1.cif

Structure factors: contains datablock(s) I. DOI: 10.1107/S160053681302148X/is5296Isup2.hkl

Supplementary material file. DOI: 10.1107/S160053681302148X/is5296Isup3.cml

Additional supplementary materials:  crystallographic information; 3D view; checkCIF report

## Figures and Tables

**Table 1:** Hydrogen-bond geometry (Å, °) *Cg*1 and *Cg*2 are the centroids of the C1–C6 and C10–C15 rings, respectively.

*D*—H⋯*A*	*D*—H	H⋯*A*	*D*⋯*A*	*D*—H⋯*A*
O2—H2*A*⋯N2	0.82	1.91	2.635 (3)	146
O1—H1⋯N1	0.82	1.93	2.646 (3)	145
C5—H5⋯*Cg*1^i^	0.93	2.84	3.519 (3)	130
C14—H14⋯*Cg*2^ii^	0.93	2.85	3.519 (3)	130

Symmetry codes: (i) 
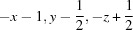
; (ii) 
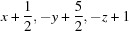
.

**Fig. 1.**
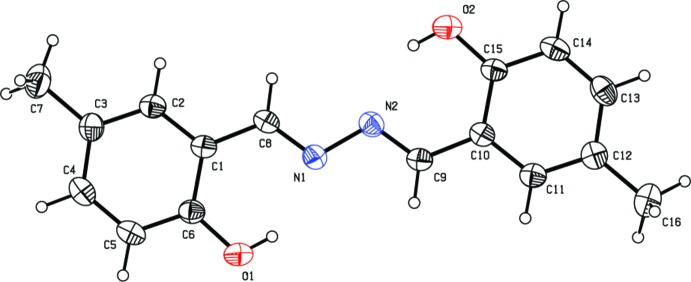
The molecular structure of the title compound, with atom labels and 30% probability displacement ellipsoids for non-H atoms.

## supplementary crystallographic information

### 1. Comment

Schiff base ligands exhibit anti-cancer, anti-fungal, anti-tumour and anti-HIV activities (Pandeya *et al.*, 1999; Singh & Dash, 1988; Kelley *et al.*, 1995; Tarafder *et al.*, 2002). In the molecular structure of the title compound (Fig. 1), the bond distances are within the normal range (Allen *et al.*, 1987) and are comparable with the related structures (Chantrapromma *et al.*, 2010; Fun *et al.*, 2010). In the molecule, two aromatic rings are almost co-planar, with a dihedral angle of 1.82 (12)°. The hydroxy groups form intramolecular O—H···N hydrogen bonds (O1—H1···N1 and O2—H2A···N2; Table 1) with S(6) graph-set motifs (Bernstein *et al.*, 1995). The crystal structure also exhibits weak intermolecular C—H···π (Table 1) interactions which forms a three dimensional network.

### 2. Experimental

The title compound was synthesized by mixing a solution (1:2 molar ratio) of hydrazine hydrate (0.20 ml, 4 mmol) and 2-hydroxy-5-methylbenzaldehyde (1.08 g, 8 mmol) in ethanol (30 ml). The resulting solution was refluxed for 4 h, yielding (65%) the pale yellow crystalline solid. The resultant solid was filtered off and washed with methanol. Pale Yellow single crystals of the title compound suitable for X-ray structure determination were recrystalized from dimethylformamide by slow evaporation at room temperature over several days.

### 3. Refinement

H atoms were positioned geometrically with C—H = 0.93–0.96 Å and O—H = 0.82 Å and allowed to ride on their parent atoms, with *U*_iso_(H) = 1.2*U*_eq_(C) or 1.5*U*_eq_(O, methyl C).

### Crystal data

**Table:**

C_16_H_16_N_2_O_2_	*F*(000) = 568
*M**_r_* = 268.31	*D*_x_ = 1.275 Mg m^−^^3^
Orthorhombic, *P*2_1_2_1_2_1_	Mo *K*α radiation, λ = 0.71073 Å
Hall symbol: P 2ac 2ab	Cell parameters from 2658 reflections
*a* = 6.0108 (5) Å	θ = 2.4–27.2°
*b* = 7.3394 (5) Å	µ = 0.09 mm^−^^1^
*c* = 31.674 (2) Å	*T* = 295 K
*V* = 1397.32 (17) Å^3^	Block, yellow
*Z* = 4	0.22 × 0.18 × 0.16 mm

### Data collection

**Table:**

Bruker Kappa APEXII diffractometer	2952 independent reflections
Radiation source: fine-focus sealed tube	1780 reflections with *I* > 2σ(*I*)
Graphite monochromator	*R*_int_ = 0.023
ω and φ scans	θ_max_ = 27.2°, θ_min_ = 2.6°
Absorption correction: multi-scan (*SADABS*; Sheldrick, 1996)	*h* = −6→7
*T*_min_ = 0.982, *T*_max_ = 0.987	*k* = −9→9
5699 measured reflections	*l* = −40→39

### Refinement

**Table:**

Refinement on *F*^2^	Primary atom site location: structure-invariant direct methods
Least-squares matrix: full	Secondary atom site location: difference Fourier map
*R*[*F*^2^ > 2σ(*F*^2^)] = 0.050	Hydrogen site location: inferred from neighbouring sites
*wR*(*F*^2^) = 0.154	H-atom parameters constrained
*S* = 1.02	*w* = 1/[σ^2^(*F*_o_^2^) + (0.0788*P*)^2^] where *P* = (*F*_o_^2^ + 2*F*_c_^2^)/3
2952 reflections	(Δ/σ)_max_ < 0.001
185 parameters	Δρ_max_ = 0.17 e Å^−^^3^
0 restraints	Δρ_min_ = −0.17 e Å^−^^3^

### Special details

**Table:**

Geometry. All esds (except the esd in the dihedral angle between two l.s. planes) are estimated using the full covariance matrix. The cell esds are taken into account individually in the estimation of esds in distances, angles and torsion angles; correlations between esds in cell parameters are only used when they are defined by crystal symmetry. An approximate (isotropic) treatment of cell esds is used for estimating esds involving l.s. planes.
Refinement. Refinement of F^2^ against ALL reflections. The weighted R-factor wR and goodness of fit S are based on F^2^, conventional R-factors R are based on F, with F set to zero for negative F^2^. The threshold expression of F^2^ > 2sigma(F^2^) is used only for calculating R-factors(gt) etc. and is not relevant to the choice of reflections for refinement. R-factors based on F^2^ are statistically about twice as large as those based on F, and R- factors based on ALL data will be even larger.

### Fractional atomic coordinates and isotropic or equivalent isotropic displacement parameters (Å^2^)

**Table:**

	*x*	*y*	*z*	*U*_iso_*/*U*_eq_	
C1	−0.1393 (4)	0.8872 (3)	0.28106 (7)	0.0424 (6)	
C2	−0.0535 (4)	0.9211 (3)	0.24097 (7)	0.0467 (6)	
H2	0.0874	0.9726	0.2387	0.056*	
C3	−0.1689 (5)	0.8812 (4)	0.20427 (8)	0.0528 (7)	
C4	−0.3790 (5)	0.8051 (4)	0.20879 (8)	0.0558 (7)	
H4	−0.4604	0.7765	0.1847	0.067*	
C5	−0.4700 (5)	0.7708 (3)	0.24753 (9)	0.0540 (7)	
H5	−0.6106	0.7185	0.2493	0.065*	
C6	−0.3559 (4)	0.8129 (4)	0.28389 (8)	0.0474 (7)	
C7	−0.0682 (6)	0.9219 (4)	0.16150 (7)	0.0766 (10)	
H7A	−0.0947	0.8212	0.1428	0.115*	
H7B	0.0891	0.9405	0.1645	0.115*	
H7C	−0.1352	1.0299	0.1500	0.115*	
C8	−0.0071 (4)	0.9275 (3)	0.31799 (7)	0.0454 (6)	
H8	0.1363	0.9726	0.3145	0.055*	
C9	−0.0019 (4)	0.9213 (3)	0.42464 (7)	0.0451 (6)	
H9	−0.1451	0.8758	0.4282	0.054*	
C10	0.1302 (5)	0.9612 (3)	0.46142 (7)	0.0429 (6)	
C11	0.0395 (5)	0.9322 (3)	0.50158 (7)	0.0478 (7)	
H11	−0.1016	0.8810	0.5035	0.057*	
C12	0.1501 (5)	0.9762 (3)	0.53841 (8)	0.0527 (7)	
C13	0.3625 (6)	1.0501 (4)	0.53396 (8)	0.0578 (8)	
H13	0.4417	1.0814	0.5581	0.069*	
C14	0.4593 (5)	1.0786 (4)	0.49519 (8)	0.0556 (7)	
H14	0.6019	1.1271	0.4935	0.067*	
C15	0.3451 (5)	1.0352 (3)	0.45871 (8)	0.0460 (6)	
C16	0.0438 (6)	0.9530 (4)	0.58087 (8)	0.0718 (9)	
H16A	−0.0435	1.0588	0.5874	0.108*	
H16B	0.1573	0.9378	0.6019	0.108*	
H16C	−0.0504	0.8474	0.5805	0.108*	
N1	−0.0818 (3)	0.9027 (3)	0.35543 (6)	0.0498 (6)	
N2	0.0716 (4)	0.9465 (3)	0.38727 (6)	0.0498 (6)	
O1	−0.4551 (3)	0.7801 (3)	0.32150 (5)	0.0680 (6)	
H1	−0.3751	0.8171	0.3406	0.102*	
O2	0.4451 (3)	1.0657 (3)	0.42094 (6)	0.0650 (6)	
H2A	0.3623	1.0328	0.4018	0.098*	

### Atomic displacement parameters (Å^2^)

**Table:**

	*U*^11^	*U*^22^	*U*^33^	*U*^12^	*U*^13^	*U*^23^
C1	0.0381 (15)	0.0357 (12)	0.0534 (14)	0.0023 (12)	−0.0003 (12)	0.0034 (10)
C2	0.0442 (15)	0.0392 (13)	0.0565 (16)	0.0000 (13)	−0.0007 (12)	0.0069 (11)
C3	0.0594 (19)	0.0491 (15)	0.0499 (15)	0.0053 (16)	−0.0034 (13)	0.0016 (12)
C4	0.0553 (19)	0.0488 (15)	0.0633 (17)	0.0011 (16)	−0.0152 (15)	−0.0051 (13)
C5	0.0412 (15)	0.0494 (14)	0.0715 (17)	−0.0039 (14)	−0.0070 (15)	−0.0016 (14)
C6	0.0417 (16)	0.0458 (14)	0.0548 (15)	0.0020 (14)	0.0002 (13)	0.0028 (11)
C7	0.102 (3)	0.076 (2)	0.0512 (16)	−0.006 (2)	−0.0028 (17)	0.0065 (14)
C8	0.0386 (16)	0.0415 (13)	0.0562 (15)	−0.0024 (13)	−0.0057 (12)	0.0003 (12)
C9	0.0395 (15)	0.0413 (13)	0.0546 (14)	−0.0010 (13)	0.0000 (12)	0.0035 (11)
C10	0.0412 (17)	0.0358 (12)	0.0518 (14)	0.0041 (12)	−0.0031 (12)	0.0002 (10)
C11	0.0465 (17)	0.0399 (13)	0.0570 (15)	0.0003 (13)	−0.0003 (13)	0.0042 (12)
C12	0.058 (2)	0.0425 (14)	0.0572 (16)	0.0059 (15)	−0.0035 (14)	0.0008 (12)
C13	0.061 (2)	0.0471 (15)	0.0656 (18)	0.0036 (16)	−0.0186 (15)	−0.0005 (13)
C14	0.0401 (17)	0.0508 (15)	0.0759 (19)	−0.0037 (15)	−0.0086 (14)	0.0047 (14)
C15	0.0379 (16)	0.0436 (14)	0.0564 (15)	−0.0008 (13)	−0.0007 (13)	0.0036 (12)
C16	0.091 (3)	0.0697 (18)	0.0550 (16)	0.005 (2)	0.0022 (17)	0.0006 (14)
N1	0.0458 (13)	0.0551 (13)	0.0485 (11)	−0.0018 (12)	−0.0044 (10)	0.0023 (10)
N2	0.0476 (13)	0.0490 (12)	0.0527 (11)	−0.0004 (11)	−0.0053 (11)	0.0021 (9)
O1	0.0460 (12)	0.0922 (16)	0.0658 (12)	−0.0134 (12)	0.0067 (10)	0.0055 (12)
O2	0.0490 (12)	0.0778 (14)	0.0683 (11)	−0.0112 (12)	0.0037 (10)	0.0053 (11)

### Geometric parameters (Å, º)

**Table:**

C1—C2	1.393 (3)	C9—H9	0.9300
C1—C6	1.415 (3)	C10—C11	1.400 (3)
C1—C8	1.445 (3)	C10—C15	1.404 (3)
C2—C3	1.385 (3)	C11—C12	1.381 (3)
C2—H2	0.9300	C11—H11	0.9300
C3—C4	1.388 (4)	C12—C13	1.395 (4)
C3—C7	1.514 (3)	C12—C16	1.499 (3)
C4—C5	1.367 (4)	C13—C14	1.375 (4)
C4—H4	0.9300	C13—H13	0.9300
C5—C6	1.376 (3)	C14—C15	1.381 (3)
C5—H5	0.9300	C14—H14	0.9300
C6—O1	1.354 (3)	C15—O2	1.357 (3)
C7—H7A	0.9600	C16—H16A	0.9600
C7—H7B	0.9600	C16—H16B	0.9600
C7—H7C	0.9600	C16—H16C	0.9600
C8—N1	1.281 (3)	N1—N2	1.404 (3)
C8—H8	0.9300	O1—H1	0.8200
C9—N2	1.277 (3)	O2—H2A	0.8200
C9—C10	1.440 (3)		
			
C2—C1—C6	117.9 (2)	C10—C9—H9	119.0
C2—C1—C8	119.9 (2)	C11—C10—C15	118.2 (2)
C6—C1—C8	122.3 (2)	C11—C10—C9	119.3 (3)
C3—C2—C1	122.8 (2)	C15—C10—C9	122.5 (2)
C3—C2—H2	118.6	C12—C11—C10	123.0 (3)
C1—C2—H2	118.6	C12—C11—H11	118.5
C2—C3—C4	117.0 (2)	C10—C11—H11	118.5
C2—C3—C7	120.6 (3)	C11—C12—C13	116.5 (3)
C4—C3—C7	122.4 (3)	C11—C12—C16	121.8 (3)
C5—C4—C3	122.0 (3)	C13—C12—C16	121.7 (3)
C5—C4—H4	119.0	C14—C13—C12	122.5 (3)
C3—C4—H4	119.0	C14—C13—H13	118.8
C4—C5—C6	120.7 (3)	C12—C13—H13	118.8
C4—C5—H5	119.6	C13—C14—C15	120.1 (3)
C6—C5—H5	119.6	C13—C14—H14	119.9
O1—C6—C5	118.5 (2)	C15—C14—H14	119.9
O1—C6—C1	122.0 (2)	O2—C15—C14	118.6 (3)
C5—C6—C1	119.5 (2)	O2—C15—C10	121.7 (2)
C3—C7—H7A	109.5	C14—C15—C10	119.7 (2)
C3—C7—H7B	109.5	C12—C16—H16A	109.5
H7A—C7—H7B	109.5	C12—C16—H16B	109.5
C3—C7—H7C	109.5	H16A—C16—H16B	109.5
H7A—C7—H7C	109.5	C12—C16—H16C	109.5
H7B—C7—H7C	109.5	H16A—C16—H16C	109.5
N1—C8—C1	121.8 (2)	H16B—C16—H16C	109.5
N1—C8—H8	119.1	C8—N1—N2	113.7 (2)
C1—C8—H8	119.1	C9—N2—N1	113.9 (2)
N2—C9—C10	122.0 (2)	C6—O1—H1	109.5
N2—C9—H9	119.0	C15—O2—H2A	109.5
			
C6—C1—C2—C3	−1.5 (4)	C15—C10—C11—C12	1.3 (4)
C8—C1—C2—C3	178.2 (2)	C9—C10—C11—C12	−176.3 (2)
C1—C2—C3—C4	0.3 (4)	C10—C11—C12—C13	−0.9 (4)
C1—C2—C3—C7	179.6 (2)	C10—C11—C12—C16	176.6 (2)
C2—C3—C4—C5	0.2 (4)	C11—C12—C13—C14	0.0 (4)
C7—C3—C4—C5	−179.1 (3)	C16—C12—C13—C14	−177.6 (2)
C3—C4—C5—C6	0.7 (4)	C12—C13—C14—C15	0.6 (4)
C4—C5—C6—O1	178.5 (2)	C13—C14—C15—O2	179.7 (2)
C4—C5—C6—C1	−1.9 (4)	C13—C14—C15—C10	−0.3 (4)
C2—C1—C6—O1	−178.2 (2)	C11—C10—C15—O2	179.4 (2)
C8—C1—C6—O1	2.1 (4)	C9—C10—C15—O2	−3.1 (4)
C2—C1—C6—C5	2.3 (4)	C11—C10—C15—C14	−0.6 (3)
C8—C1—C6—C5	−177.4 (2)	C9—C10—C15—C14	176.9 (2)
C2—C1—C8—N1	178.2 (2)	C1—C8—N1—N2	179.0 (2)
C6—C1—C8—N1	−2.0 (4)	C10—C9—N2—N1	−179.07 (19)
N2—C9—C10—C11	179.4 (2)	C8—N1—N2—C9	−179.7 (2)
N2—C9—C10—C15	2.0 (4)		

### Hydrogen-bond geometry (Å, º)

*Cg*1 and *Cg*2 are the centroids of the C1–C6 and C10–C15 rings, respectively.

**Table:**

*D*—H···*A*	*D*—H	H···*A*	*D*···*A*	*D*—H···*A*
O2—H2*A*···N2	0.82	1.91	2.635 (3)	146
O1—H1···N1	0.82	1.93	2.646 (3)	145
C5—H5···*Cg*1^i^	0.93	2.84	3.519 (3)	130
C14—H14···*Cg*2^ii^	0.93	2.85	3.519 (3)	130

Symmetry codes: (i) −*x*−1, *y*−1/2, −*z*+1/2; (ii) *x*+1/2, −*y*+5/2, −*z*+1.
